# Analysis of DNA Repair-Related Prognostic Function and Mechanism in Gastric Cancer

**DOI:** 10.3389/fcell.2022.897096

**Published:** 2022-05-17

**Authors:** Liqiang Wang, Jianping Lu, Ying Song, Jing Bai, Wenjing Sun, Jingcui Yu, Mengdi Cai, Songbin Fu

**Affiliations:** ^1^ Key Laboratory of Preservation of Human Genetic Resources and Disease Control in China (Harbin Medical University), Ministry of Education, Harbin, China; ^2^ Laboratory of Medical Genetics, Harbin Medical University, Harbin, China; ^3^ College of Bioinformatics Science and Technology, Harbin Medical University, Harbin, China; ^4^ Scientific Research Centre, The Second Affiliated Hospital of Harbin Medical University, Harbin, China

**Keywords:** gastric cancer, DNA repair, tumor mutational burden, immune infiltration, DNA methylation, prognostic model

## Abstract

DNA repair mechanisms have been proven to be essential for cells, and abnormalities in DNA repair could cause various diseases, such as cancer. However, the diversity and complexity of DNA repair mechanisms obscure the functions of DNA repair in cancers. In addition, the relationships between DNA repair, the tumor mutational burden (TMB), and immune infiltration are still ambiguous. In the present study, we evaluated the prognostic values of various types of DNA repair mechanisms and found that double-strand break repair through single-strand annealing (SSA) and nonhomologous end-joining (NHEJ) was the most prognostic DNA repair processes in gastric cancer (GC) patients. Based on the activity of these two approaches and expression profiles, we constructed a HR-LR model, which could accurately divide patients into high-risk and low-risk groups with different probabilities of survival and recurrence. Similarly, we also constructed a cancer-normal model to estimate whether an individual had GC or normal health status. The prognostic value of the HR-LR model and the accuracy of the cancer-normal model were validated in several independent datasets. Notably, low-risk samples, which had higher SSA and NHEJ activities, had more somatic mutations and less immune infiltration. Furthermore, the analysis found that low-risk samples had higher and lower methylation levels in CpG islands (CGIs) and open sea regions respectively, and had higher expression levels of programmed death-ligand 1 (PD-L1) and lower methylation levels in the promoter of the gene encoding PD-L1. Moreover, low-risk samples were characterized primarily by higher levels of CD4^+^ memory T cells, CD8^+^ naive T cells, and CD8^+^ TEM cells than those in high-risk samples. Finally, we proposed a decision tree and nomogram to help predict the clinical outcome of an individual. These results provide an improved understanding of the complexity of DNA repair, the TMB, and immune infiltration in GC, and present an accurate prognostic model for use in GC patients.

## Introduction

Gastric cancer (GC) is the sixth most prevalent cancer in the world and the third leading cause of cancer-related deaths (Bray et al., 2018). Despite remarkable progress in diagnostic and therapeutic methods, GC remains a refractory malignancy. In recent years, significant progress has been made in understanding cancer-associated molecular genetics as a result of the development of molecular biology methods. Several studies have found that mutations in the genome play an indispensable role in genomic maintenance and evolution. Furthermore, studies have demonstrated that when DNA repair mechanisms are disrupted or deregulated this may increase rates of mutagenesis and genomic instability and thereby mediate cancer progression (Bouwman and Jonkers, 2012; Wolters and Schumacher, 2013). The main DNA repair mechanisms include direct repair, base excision repair, and double-strand break repair, among which DNA double-strand break (DSB) repair plays a crucial role in maintaining genomic integrity (Gillyard and Davis, 2021). In addition, DSB affects the prognosis of cancer by preventing disadvantageous mutations (Stok et al., 2021). However, it remains unknown whether DSB is valuable for predicting the clinical outcomes of GC patients.

The tumor mutational burden (TMB) refers to the number of somatic mutations per DNA megabase in tumor cells. TMB is considered as the primary driver of antitumor adaptive immune responses and serves as a positive predictive biomarker for immune checkpoint inhibitors (Castle et al., 2012). Recently, various studies have illustrated that the genomic instability resulting from the inadequacy of DNA repair mechanisms is associated with high TMBs. Preclinical studies identified that tumor cells with higher TMB could produce more neoantigens, which are more easily recognized by T cells, and thus activate stronger immune killing activity (Parikh et al., 2019; Klempner et al., 2020). However, there are some limitations to the use of the TMB as a biomarker for clinical utilization. This is mainly because heterogeneity among intratumoral neoantigens may also be important for immunotherapy response (McGranahan et al., 2016). Furthermore, it is also important to acknowledge that the cut-off values of TMB lack standardization and consistency because they are defined differently across studies, testing platforms, and patient populations (Gibney et al., 2016). Thus, the development of predictive biomarkers is urgently needed to benefit patients. In this situation, an increasing number of oncologists have begun to focus their studies on PD-L1 expression of tumor cells and found that PD-L1 positivity has emerged as a major predictive marker (Borghaei et al., 2015; Larkin et al., 2015). However, the accuracy is not satisfactory based on PD-L1 as a single molecule for GC patients (Patel and Kurzrock, 2015). This is mainly because the expression of PD-L1 is also affected by other factors. For example, its expression is associated with global hypomethylation (Emran et al., 2019). To improve clinical outcomes in GC, it is essential to explore emerging biomarkers through a comprehensive multifaceted analysis involving DNA repair mechanisms, tumor-infiltrating lymphocytes, TMB, mutational signatures, immune microenvironments of tumors, and immune checkpoints.

In the present study, we constructed a HR-LR model to improve the performance of the prognosis of overall survival and recurrence of GC patients by integrating the DNA repair-related GO processes from the MsigDB database and the clinical data from TCGA stomach adenocarcinoma (GC) patients. Moreover, we estimated the expression level of PD-L1 as an immune checkpoint and methylation level of CpG sites in the PD-L1 promoter region, TMB, and the systemic immune status. To a certain extent, attempting to exploit a novel model based on DNA repair mechanisms will significantly help select GC patients who would benefit from predictions of clinical outcomes and improve the accuracy of prognostic assessments.

## Materials and Methods

### Transcriptomic, Genomic, and Clinical Datasets of the Cancer Genome Atlas Cohort

Transcriptional profiles of cancer and normal tissues of patients with GC were obtained from stomach adenocarcinoma (STAD) patients of The Cancer Genome Atlas (TCGA, https://portal.gdc.cancer.gov), including 375 cancer samples and 32 normal samples (Table 1). For cancer or normal samples, genes with FPKM expression values of 0 in >70% of samples were removed, and the remaining 0 values were imputed with K-Nearest Neighbors. Then, expression values were log2 transformed for subsequent analysis.

**TABLE 1:** Patient cohorts from TCGA and GEO databases.

Cohort	Cancer samples	Normal samples	Recurrence	GPL
TCGA	375^a^ (242 training +106 test)	32	r^b^	-
GSE62254	300	-	R	GPL570
GSE26253	432	-	R	GPL8432
GSE84437	433	-	-	GPL6947
GSE26899	96	-	R	GPL6947
GSE15460	248^c^	-	-	GPL570
GSE13861	65	25	r	GPL6884
GSE13911	38	31	-	GPL570
GSE33335	25	25	-	GPL5175
GSE66229	300 (GSE62254)	100	-	GPL570

a In total, we obtained 375 cancer and 32 normal samples from TCGA, database. Screening samples with survival data and deleting those samples died in 10 days, we finally obtained 348 samples. Then we selected 242 samples as the training set randomly while the remaining 106 samples were as the test set.

b “r” in the table represented the datasets with recurrence information.

c The dataset GSE15460 included GSE15455, GSE15456, GSE15459, GSE15537, GSE22183, GSE34942 datasets. Deleting those cell line datasets and the datasets sequenced by GPL96, we finally obtained 248 samples, including GSE15459 and GSE34942.

Mutational data of GC patients was also downloaded from the TCGA database. After removing the synonymous variants, we calculated the tumor mutational burden (TMB), which was defined as the number of somatic mutations per megabase of interrogated genomic sequence. Mutation profiles were analyzed and visualized by the R package “maftools” (Mayakonda et al., 2018).

We obtained clinical information of GC patients from the TCGA database, including survival state, survival time, recurrence state, recurrence time, disease stage, therapeutic response, age, gender, and other clinical characteristics.

### DNA Repair Related GO Terms and Pathways

The genes of GO terms and pathways related to DNA repair mechanism were downloaded from the Molecular Signatures Database (MsigDB) (Liberzon et al., 2015). Based on the transcriptional data of these genes in the TCGA cohort, we calculated the score of each GO term or pathway using a single-sample gene set enrichment analysis (ssGSEA). A univariate Cox proportional-hazards regression model was used to evaluate the prognostic significance of each GO term or pathway in GC by R package “survival”. Taken the results of DNA repair-related GO terms, KEGG pathways, and Reactome pathways, double-strand break repair through single-strand annealing (SSA) or nonhomologous end-joining (NHEJ) were the most prognostic approaches. The result was validated in GSE66254 (GPL570) dataset.

The ssGSEA score of SSA or NHEJ GO term in normal samples and good or poor outcome samples were also calculated by the ssGSEA algorithm. Those samples that lived more than 1 year were defined as good outcome samples while dead samples in 1 year were defined as poor outcome samples.

### Construction of HR-LR Model

We proposed a computational method to establish the HR-LR model, which involved three steps. First, we randomly divided the STAD TCGA cohort into training and test sets, including 242 and 106 samples, respectively (Table 1). The HR-LR model was established based on the training set. Second, we selected those genes with significant associations with a score of SSA and NHEJ GO terms as the DNA repair-related marker genes (|*R*| > 0.4, *BH-FDR* < 0.001). In total, we obtained 37 positive genes and 39 negative genes. Third, an SSA-NHEJ score was defined by the *T* statistic of a two-sided *t*-test for each tumor sample by comparing the expression values of the 37 positively correlated genes with the expression values of the 39 negatively correlated genes (Table 2 and Supplementary Table S1).

**TABLE 2:** Positive and negative genes used in the HR-LR model and Cancer-Normal model.

	Gene symbols
Positive genes	BRIP1, CDC45, CDC7, CDCA2, CENPK, CLSPN, DDIAS, DLGAP5, DTL, E2F7, EZH2, FANCA, HELLS, HIST1H2AH, KIF11, KIF15, KIF18A, KIF23, KIF2C, KNTC1, LMNB1, MCM10, MND1, NCAPG, ORC1, PCNA, PLK4, POLE2, POLQ, POLR3G, RAD51AP1, RAD54L, RFC4, RRM2, TYMS, UHRF1, XRCC2
Negative genes	ADCY5, APOD, C15orf59, C16orf89, C1QTNF2, C1QTNF7, CGNL1, CRYAB, DAAM2, DACT3, DCN, ELN, FAM110B, FMOD, GHR, GREM2, GSTM5, HSPA2, HSPB8, KCNK3, LRRN4CL, MFAP4, NDNF, NEGR1, NFATC4, PDE2A, PPP1R14A, PPP1R3C, SAMD11, SCN4B, SCUBE2, SLC22A17, SMARCD3, SRPX, TCEAL7, TMEM100, TMOD1, TNFAIP8L3, ZCCHC24

The median SSA-NHEJ score of training samples was defined as the cutoff (cutoff = 3.46). An SSA-NHEJ score >3.46 represented that those 37 positive genes were overexpressed while the 39 negative genes were underexpressed. An SSA-NHEJ score <3.46 meant the opposite. As SSA and NHEJ GO terms were protective factors in GC, patients with higher SSA-NHEJ scores were considered with a better outcome. Therefore, the samples were divided into high-risk and low-risk groups, with low and high SSA-NHEJ scores, respectively. The survival hypothesis was validated in training, test sets of the TCGA cohort, and other GEO cohorts. In addition, we found that those high-risk samples were more likely to be recurrent, compared with those low-risk samples.

### Construction of Cancer-Normal Model

The model was established exactly like the HR-LR model. However, the cutoff was changed. Using the R package “pROC” (Robin et al., 2011), we selected 0.008 as the cutoff. Samples with SSA-NHEJ score ≥0.008 were predicted as tumor samples while other samples were predicted as normal samples.

### Transcriptomic and Clinical Datasets of GEO Validation Cohorts

The independent validation datasets were downloaded from the Gene Expression Omnibus database, including nine datasets. The detailed information is shown in Table 1. We chose six datasets with expression profiles and survival information to validate our HR-LR model, including GSE62254, GSE26253, GSE84437, GSE26899, GSE15460, and GSE13861. For validation of GC recurrence, we selected four datasets with recurrence information, including GSE62254, GSE26253, GSE13861, and GSE26899. Finally, we chose the datasets with cancer and normal samples to validate the cancer-normal model, including GSE13861, GSE13911, GSE33335, and GSE66229.

### Generation of ImmuneScore, StromalScore, EstimateScore, and MicroenvironmentScore

For each patient sample, ImmuneScore, StromalScore, EstimateScore, and MicroenvironmentScore were generated by R package “estimate” (Yoshihara et al., 2013) and R package “xCell” (Aran et al., 2017). The higher the respective score, the larger the ratio of the corresponding component in the tumor microenvironment (TME). The infiltration of immune and stromal cell types in an individual sample was evaluated by R package “xCell”.

### Calculation of DNA Hyper- and Hypomethylation Scores in Tumor Samples

DNA methylation dataset of STAD patients detected by Illumina Infinium HumanMethylation450 BeadChip array was also downloaded from the TCGA data portal. After selecting the promoter CpG islands (CGIs) and open sea regional clusters on the genome, we calculated the aberrant hypermethylation (over CGI probes) and hypomethylation (over open sea probes) values for each tumor sample compared with normal samples according to one of the previous studies (Yang et al., 2015). The scores of hyper- and hypomethylation were calculated as follows: 1) all genome CpG sites were classified into different regional classes, then CGI and open sea regions were selected to be grouped into regional clusters by boundedClusterMaker in R-package bumphunter (maximum cluster width of 1500bp and maximum gap of 500bp between any two neighboring regional classes), respectively; 2) the methylation in each cluster was defined as the mean beta value of the sites within the cluster; 3) for each cluster in a certain cancer sample, the relative methylation was calculated as the beta value of this cluster in single cancer sample subtracting the mean value and further dividing the standard value of beta value of this cluster in all normal samples; 4) since, cluster regions in promoter CGIs and open sea usually show hypermethylated and hypomethylated in cancer samples, we calculated the hyper- and hypomethylation for a cancer sample as the mean of positive and negative relative methylation value in all region clusters, respectively.

### Survival Analysis

Kaplan–Meier survival plots and log-rank tests were used to evaluate the survival differences between groups of patients. The univariate Cox proportional-hazards regression model and multivariate Cox proportional-hazards regression model were used to evaluate the prognostic significance of factors. This process was performed using the R package “survival”.

### Decision Tree

Combining the HR-LR model and patient stage information, a decision tree was established to predict the single sample into low-risk, moderate-risk, and high-risk groups. The relationship between predicted results and the sample real tags was plotted as a Sankey diagram, using the R package “networkD3”.

### Nomogram Plot

A nomogram was built with SSA-NHEJ score and other clinical features to quantify the risk assessment for the individual patient, using the R package “rms” (Zhang and Kattan, 2017).

## Results

### The Single-Strand Annealing and Nonhomologous End-Joining DNA Repair Approaches ere Identified as the Primary Predictive Factors for Overall Survival in Gastric Cancer Patients

**FIGURE 1:**
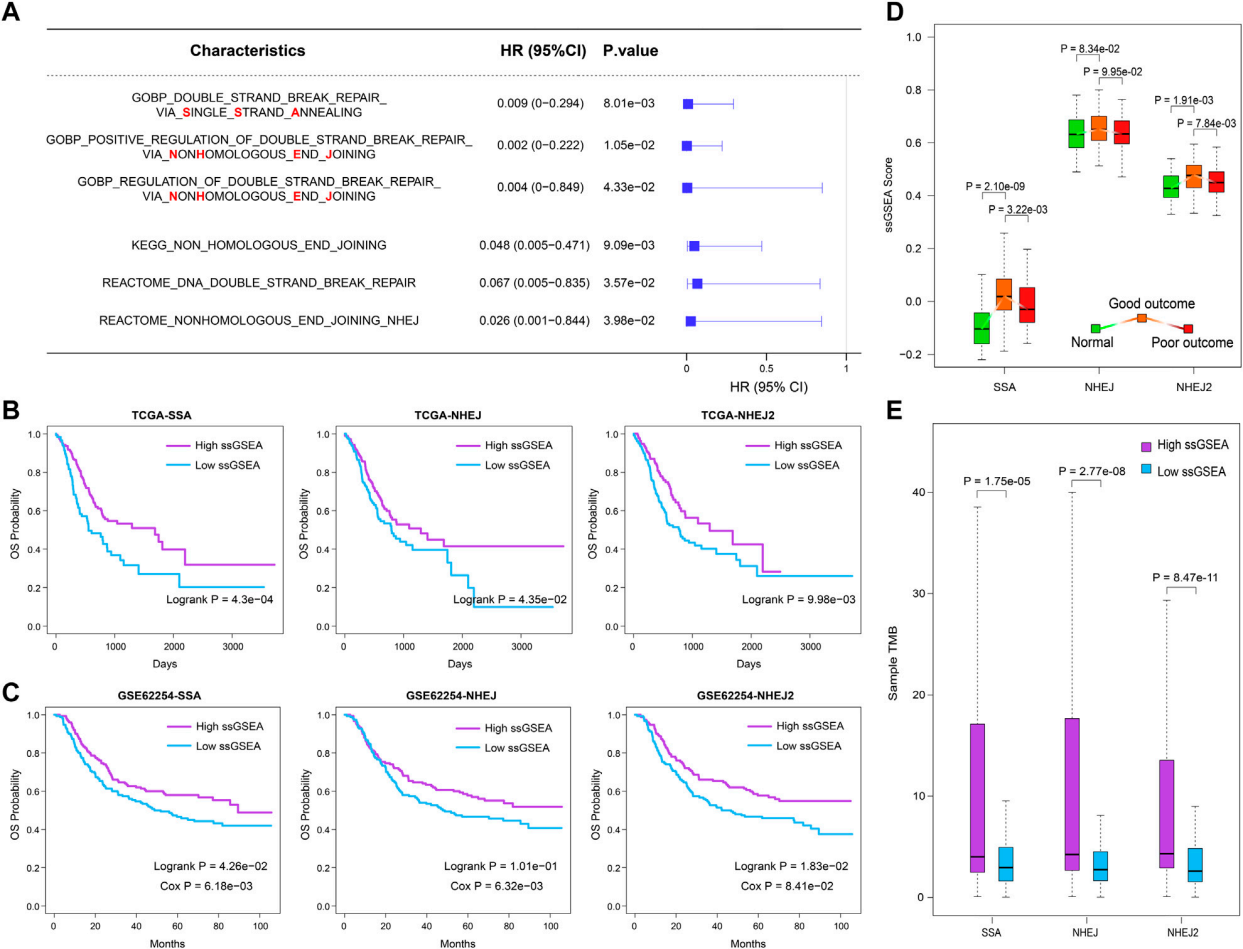
SSA and NHEJ DNA repair approaches are primary protection factors for overall survival in GC patients. **(A)** The prognostic value of SSA and NHEJ DNA repair process evaluated by univariate Cox proportional-hazards regression model. **(B,C)** Samples with higher ssGSEA scores of SSA or NHEJ have better clinical outcomes in the TCGA cohort and GSE62254 dataset. **(D)** ssGESA scores of SSA and NHEJ processes increase first and then decline in normal, good outcome, and poor outcome samples. **(E)** Samples with a higher ssGSEA score of SSA or NHEJ are with more somatic mutations.

On the basis of the expression profiles of the TCGA STAD cohort and the gene lists extracted from the MSigDB, we calculated the performance score (ssGSEA score) for each DNA repair-related GO term, KEGG pathway, and Reactome pathway (Supplementary Table S2). The univariate Cox coefficient and significance of each GO term and pathway were determined. Summarizing the results, we found that DSB repair was the most effective prognostic factor (Supplementary Figures S1A–C). Among the DSB repair mechanisms, SSA and NHEJ were found to be the primary predictive factors for outcomes in the TCGA STAD cohort (Figure 1A). Because DNA repair mechanisms are described in more detail by GO terms, we then selected “double-strand break repair *via* single-strand annealing,” “positive regulation of double-strand break repair *via* nonhomologous end joining,” and “regulation of double-strand break repair *via* nonhomologous end joining,” for subsequent analysis (HR = 0.009, 0.002, and 0.004, respectively; *p* = 8.01e−03, 1.05e−02 and 4.33e−02, respectively). Kaplan–Meier survival plots showed that patients with higher ssGSEA scores have better outcomes (Figure 1B; log-rank *p* = 4.3e−04, 4.35e−02, and 9.98e−03, respectively). These results were validated in the independent GSE62254 dataset (Figure 1C). This dataset was selected because there are relatively more genes corresponding to the three abovementioned GO terms in data from the GPL570 platform.

To examine the performance of these three prognostic DNA repair-related GO terms, we then compared their ssGSEA scores between three groups from the TCGA cohort, namely, normal samples, good-outcome samples, and poor-outcome samples. Here, we defined the good-outcome samples as those patients who had a survival time of greater than 1 year, while those patients who died within 1 year were regarded as poor-outcome samples. The results showed that the ssGSEA scores of SSA and NHEJ were the lowest in normal samples (Figure 1D). This suggests that the SSA and NHEJ repair patterns have higher activity in cancer cells in comparison with normal cells, which may be caused by the fact that more DNA replication and more errors occur in cancer cells. On the other hand, among cancer samples, good-outcome samples had significantly higher ssGSEA scores in comparison with poor-outcome samples (Figure 1D). This result was consistent with the result of univariate Cox regression as shown in Figure 1A. Because repair by SSA and NHEJ could lead to an accumulation of mutations, we then calculated the TMB for each cancer sample and compared the TMBs of samples with high and low ssGSEA scores. As was expected, samples with higher ssGSEA scores had significantly higher TMBs (*p* = 1.75e−05, 2.77e−08, and 8.47e−11 for SSA, NHEJ, and NHEJ2, respectively; Figure 1E).

Combining these results, we suggest that the SSA and NHEJ repair mechanisms were enhanced when normal cells were converted into cancer cells and that those patients with stronger SSA and NHEJ repair mechanisms had more mutations, which may have resulted in the production of more new antigens, and thus finally had better outcomes.

### The HR-LR Model Is a Highly Effective Prognostic Factor for Gastric Cancer Patients

**FIGURE 2:**
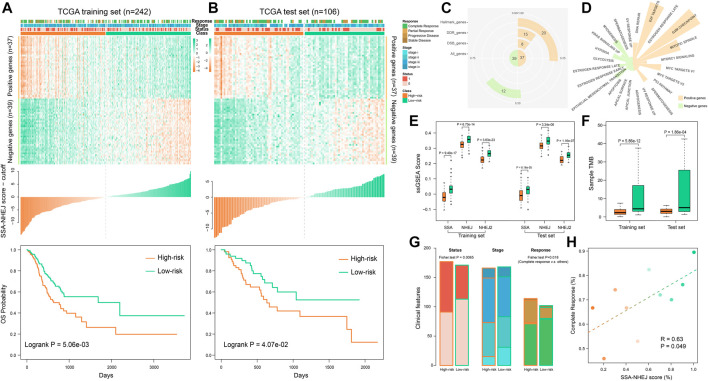
Using HR-LR model to predict survival risk of samples. **(A)** Top panel shows the expression profile of the SSA and NHEJ-related marker genes in the TCGA training set. The middle panel shows the score of each training sample by the HR-LR model. The bottom panel represents the Kaplan–Meier survival plot of high-risk and low-risk samples in the training set. **(B)** Expression profile, sample score, and Kaplan–Meier survival plot for TCGA test samples. **(C)** The statistic of 76 marker genes. **(D)** Marker genes related to cancer hallmarks. **(E)** Low-risk samples are with significantly higher SSA and NHEJ ssGSEA scores in training and test sets. **(F)** Low-risk samples have significantly more somatic mutations. **(G)** Low-risk samples are with more alive, low stage, and complete response samples. **(H)** Correlation of SSA-NHEJ score with complete response. Samples were divided into ten groups according to their scores. Samples with higher scores are with a higher probability of complete response.

We developed the HR-LR model in three steps, as described in the Materials and Methods section. Using the HR-LR model, we calculated the SSA-NHEJ score for each sample in the training and test sets. Kaplan–Meier survival plots were generated and log-rank tests were executed for samples with high- and low- SSA-NHEJ scores (with the median score as the cut-off) in the training set (*n* = 242). As a result, we found that samples with higher SSA-NHEJ scores had better clinical outcomes (log-rank *p* = 5.06e−03; Figure 2A). Then, we validated the predictive effect of the SSA-NHEJ score in the test set (*n* = 106, log-rank *p* = 4.07e−02; Figure 2B). The cut-off remained unchanged in the test set.

To characterize the functions of genes in the HR-LR model, a functional statistical analysis was performed on them. Among the 37 positively correlated genes, 8, 15, and 20 genes were associated with DSB repair, DNA repair, and cancer hallmarks, respectively. Of the 39 negatively correlated genes, 12 were associated with cancer hallmarks (Figure 2C, Table 2, and Supplementary Table S1). Furthermore, we have listed the associated cancer hallmarks in detail in Figure 2D, including several well-known examples such as DNA repair, apoptosis, hypoxia, glycolysis, the epithelial–mesenchymal transition, the p53 pathway, and KRAS signaling (Supplementary Table S1). These results suggested that these 76 genes were associated with not only DNA repair by SSA and NHEJ but also significant biological mechanisms in the body and, thus, ultimately affected the development of GC and clinical outcomes in patients.

Comparing the performance of the SSA and NHEJ GO terms, we found that high-risk samples had significantly lower ssGSEA scores in both the training set and the test set (Figure 2E). Furthermore, we found that high-risk samples had significantly lower TMB or fewer somatic mutations (Figure 2F). These results were consistent with previous studies, as samples with higher TMBs had better outcomes owing to the generation of more new antigens (Parikh et al., 2019; Klempner et al., 2020). Analyzing the clinical features of samples, we found that high-risk samples had more deaths, higher disease stages, and poor responses to therapy (Figure 2G). The probability of patients achieving complete response was significantly correlated with the SSA-NHEJ score (Figure 2H; Pearson correlation coefficient r = 0.63, *p* = 0.049).

**FIGURE 3:**
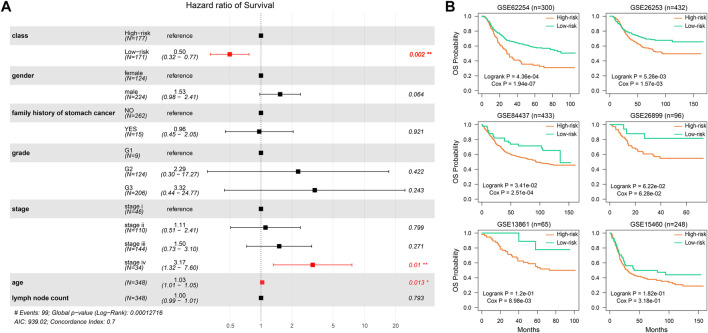
The prognostic effect of the HR-LR model. **(A)** Multivariate Cox proportional-hazards regression result of SSA-NHEJ score and other clinical characteristics. **(B)** Validation of the prognostic effect of the SSA-NHEJ score in six GEO cohorts. *n* represents the number of samples in each GEO dataset.

To validate the prognostic effect of the SSA-NHEJ score, we first performed a multivariate Cox regression modeling analysis involving the SSA-NHEJ score, age, gender, disease stage and grade, lymph node count, and family history of stomach cancer. The results showed that the SSA-NHEJ score was the most effective prognostic factor (HR = 0.50 and *p* = 0.002 for low-risk samples), which indicated that the SSA-NHEJ score was an independent prognostic factor for GC patients (Figure 3A).In addition, patients with stage IV disease and older ages were found to have poor outcomes. To investigate the applicability of the HR-LR model and validate its prognostic effect, we collected several independent datasets involving GC patients from the GEO database (Table 1). Using the expression of genes in the HR-LR model and the cut-off derived from the TCGA training set in each GEO dataset, we calculated the SSA-NHEJ score for each sample and further divided the samples into high-risk and low-risk groups. The results showed that in six independent GEO datasets from different GEO platforms the HR-LR model had a significant prognostic effect (Figure 3B). These results confirmed the prognostic value of the HR-LR model and established that this model could be applied in different datasets, including RNA-Seq data and microarray data from different platforms.

### The HR-LR Model Can Also Predict Recurrence in Gastric Cancer Patients

**FIGURE 4:**
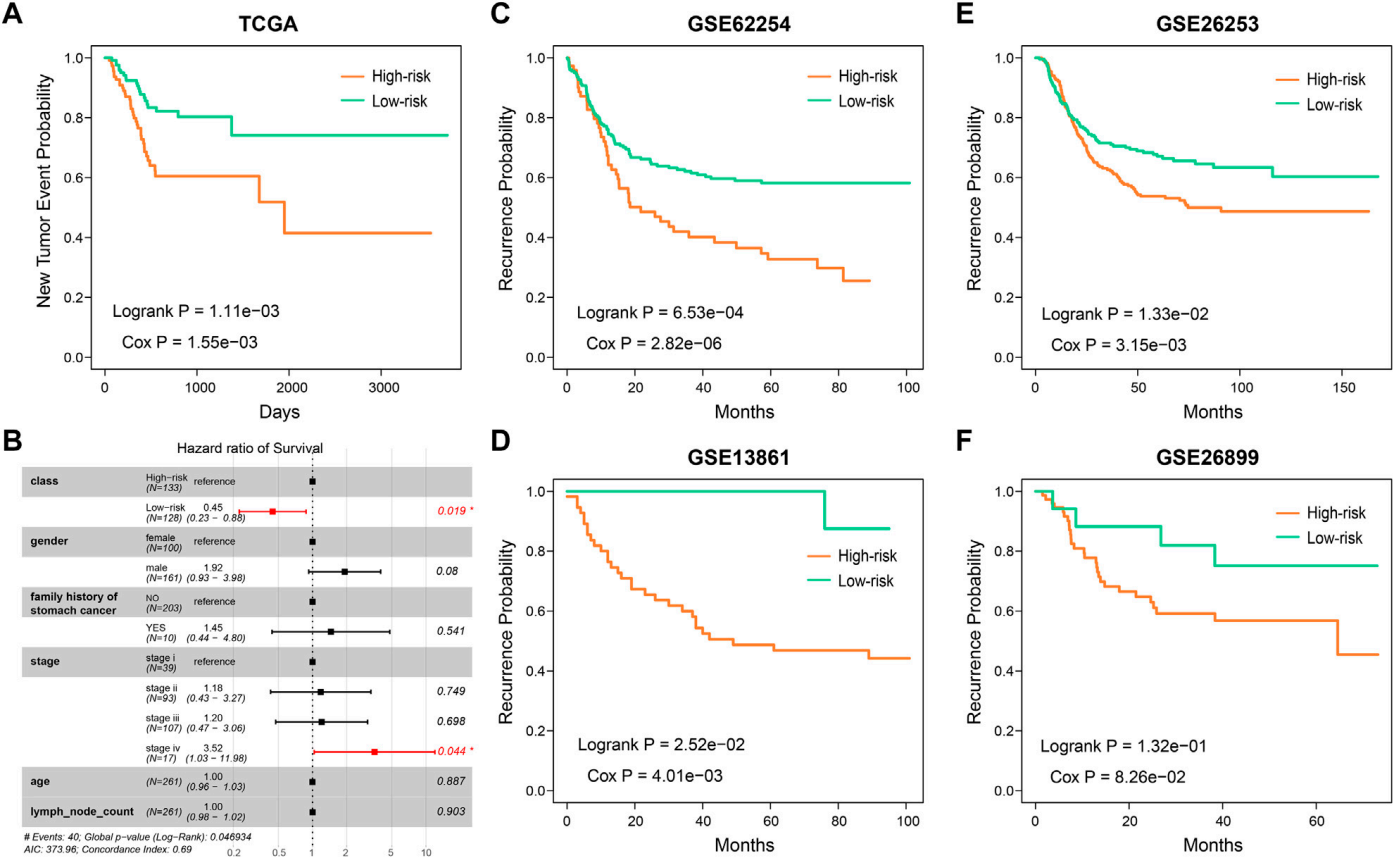
The predictive ability of SSA-NHEJ score for recurrence of GC patients. **(A)** New Tumor Event probability of patients in TCGA cohort. **(B)** Multivariate Cox proportional-hazards regression result for recurrence prognosis of the SSA-NHEJ score and other clinical characteristics. **(C–F)** Recurrence probability of patients in four GEO cohorts.

As the HR-LR model had a robust prognostic effect with regard to overall survival in GC patients, we then tested its predictive effect on recurrence in GC patients. Integrating the training and test sets, we found that the HR-LR model could divide samples into high recurrence risk and low recurrence risk groups in the TCGA cohort (Figure 4A; log-rank *p* = 1.11e−03, Cox *p* = 1.55e−03). Multivariate Cox regression modeling analysis showed that the SSA-NHEJ score was a prognostic factor for recurrence of GC that was independent of clinical features (Figure 4B; HR = 0.45 and Cox *p* = 0.019 for low-risk samples). In addition, the prognostic effect of the HR-LR model with regard to recurrence was also validated in four other GEO datasets (Figures 4C–F; log-rank *p* = 6.53e−04, 1.33e−02, 2.52e−02 and 1.32e−01 and Cox *p* = 2.82e−06, 3.15e−03, 4.01e−03 and 8.26e−02 for the GSE62254, GSE26253, GSE13861, and GSE26899 datasets, respectively).

### Low-Risk Samples Had Higher TMBs

**FIGURE 5:**
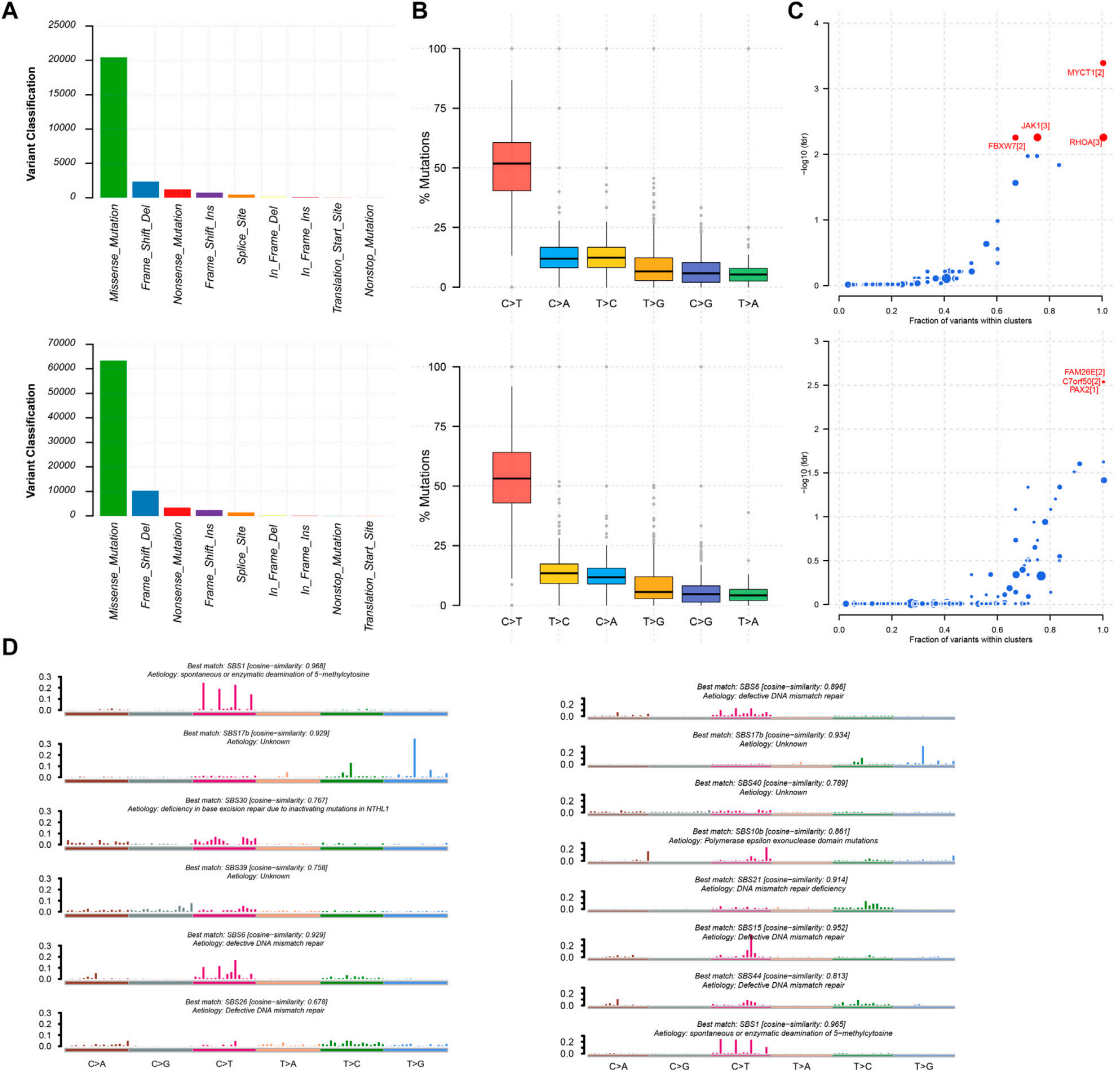
Mutation information of high-risk samples and low-risk samples. **(A,B)** Statistic of variant classification and mutation type of samples with high-risk (top panel) and low-risk (bottom panel) survival. **(C)** Driver mutation genes in high-risk and low-risk samples (top and bottom panel respectively). **(D)** Mutational signatures were identified in high-risk and low-risk samples, respectively. The plot title indicates the best match against validated COSMIC signatures (left and right panel respectively).

We utilized the maftools package to visualize the results on the basis of somatic mutation data from the TCGA STAD cohort. These somatic mutations included point mutations and insertions/deletions. An oncoplot plot showed that missense mutations occupied an absolute position among total mutations and that the number of mutations in low-risk samples was higher than that in high-risk samples (Figure 5A). Then, a transition and transversion plot was used to classify single-nucleotide variants into six categories (i.e., T > G, T > C, T > A, C > T, C > G, and C > A; Figure 5B). Moreover, it was found that C > T had the highest frequency among single-nucleotide variants in both low-risk and high-risk samples.

Cancer driver genes could provide an advantage for the selective growth of cancer cells. Therefore, we applied the OncodriveCLUST algorithm in the TCGA STAD cohort, which detected a majority of the activating mutations and identified seven well-known oncogenes as significantly mutated in 348 samples (false discovery rate <0.01). MYCT1, RHOA, JAK1, and FBXW7 were identified in high-risk samples. Likewise, we identified FAM26E, C7orf50, and PAX2 in low-risk samples (Figure 5C). Whereas the Oncodrive algorithm was more advantageous in terms of sensitivity in identifying oncogenes with mutational hotspots, in contrast, the Oncodrive algorithm underperformed in identifying potential tumor suppressor genes (Mayakonda et al., 2018). In consequence, we did not identify potential tumor suppressor genes, such as *TP53*.

As cancer progresses, a characteristic mutational pattern may be left behind at various points in time, which may reveal its underlying mutagenic process (Alexandrov et al., 2013). Therefore, we further analyzed mutational signatures on the STAD TCGA cohort by performing signature enrichment. In total, we identified six and eight signatures in high-risk and low-risk samples, respectively (Figure 5D). Interestingly, we identified the common signature associated with DNA mismatch repair in both types of samples was associated with high numbers of small (shorter than 3bp) insertions and deletions at mono/polynucleotide repeats. The deficiency in base excision repair due to inactivating mutations in NTHL1 was specific to the mutational signature of high-risk samples and primarily caused predominantly C > G mutations. This may be due to the generation of abasic sites after removal of uracil by base excision repair. In addition, polymerase epsilon exonuclease domain mutations were specific to the mutational signature of low-risk samples and exhibited strand bias for C > A mutations in the TpCpT context and T > G mutations in the TpTpT context. The mutational process underlying this signature was altered activity of the error-prone polymerase POLE. It has been proposed that the presence of large numbers of this signature was associated with recurrent POLE somatic mutations. The observations from signature enrichment suggest that DNA mismatch repair mechanisms play a crucial role in the development of malignant tumors, in accordance with previous reports (Belfield et al., 2018; McCarthy et al., 2019).

### Low-Risk Samples Were Associated With Less Immune Cell Infiltration

**FIGURE 6:**
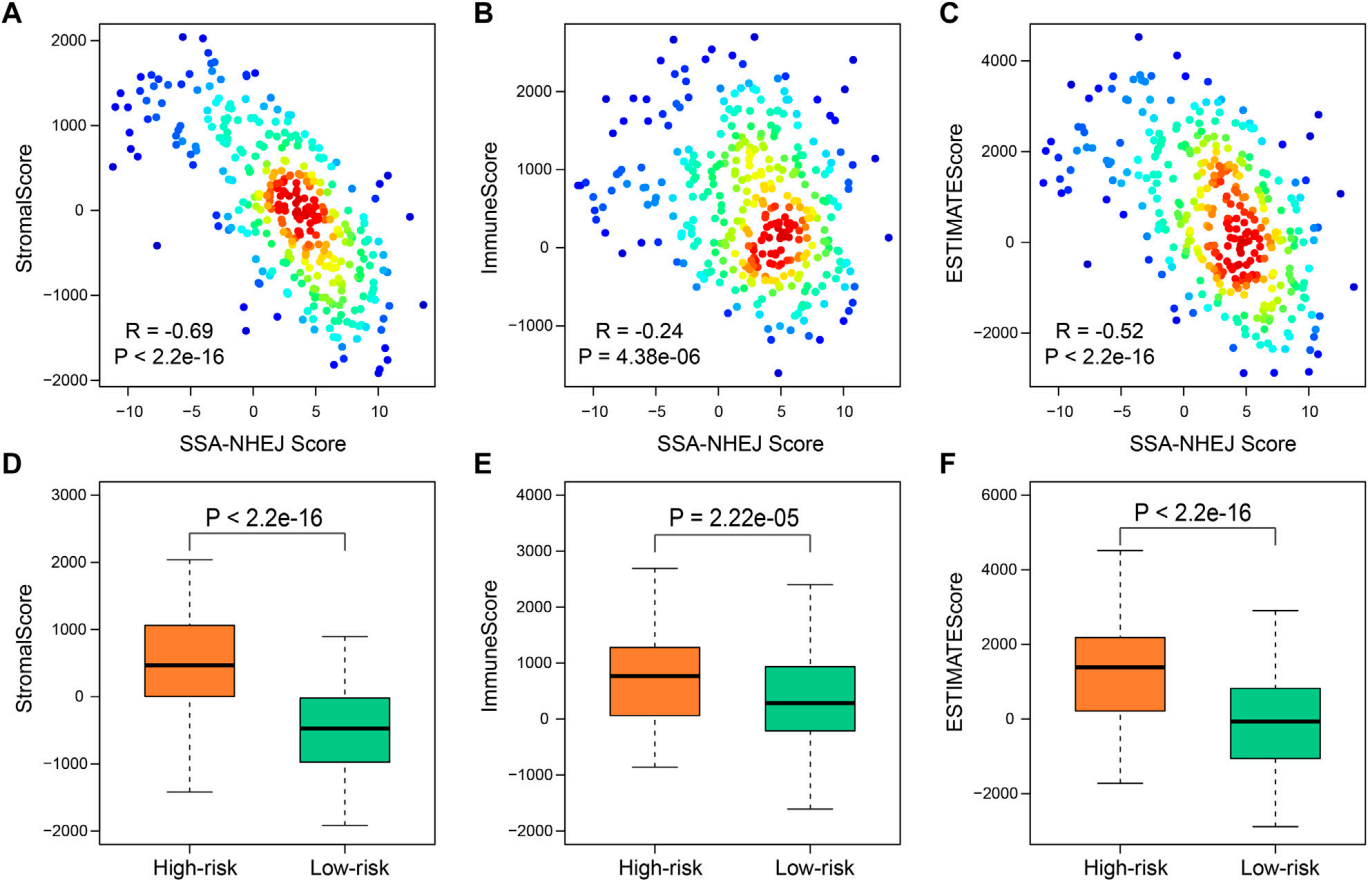
Immune-related score by “estimate”. **(A–C)** Correlation between SSA-NHEJ score and StromalScore **(A)**, ImmuneScore **(B)**, and ESTIMATEScore **(C)** in TCGA samples. R and P were calculated by the Pearson test. **(D–F)** Low-risk samples have lower StromalScore **(D)**, ImmuneScore **(E)**, and ESTIMATEScore **(F)** compared with high-risk samples. *p* values were calculated by Wilcoxon test.

To further clarify the intrinsic biological differences between high-risk and low-risk samples, the ESTIMATE algorithm was used for the estimation of stromal cells and immune cells in malignant tumors by calculating the corresponding scores. A higher immune score or stromal score represents a larger amount of immune or stromal components in the tumor microenvironment. To investigate correlations between the stromal score, immune score, and ESTIMATE score and the SSA-NHEJ score, Pearson’s correlation coefficient were used to measure the strength of the respective correlations. The results indicated that the stromal score, immune score, and ESTIMATE score were negatively correlated with the SSA-NHEJ score and decreased significantly with an increase in the SSA-NHEJ score (Figures 6A–C). We then determined the differences in the immune score, stromal score, and ESTIMATE Score between high-risk and low-risk samples. The results showed that the average immune score (Figure 6D), stromal score (Figure 6E), and ESTIMATE score (Figure 6F) were significantly higher in high-risk samples than in low-risk samples.

**FIGURE 7:**
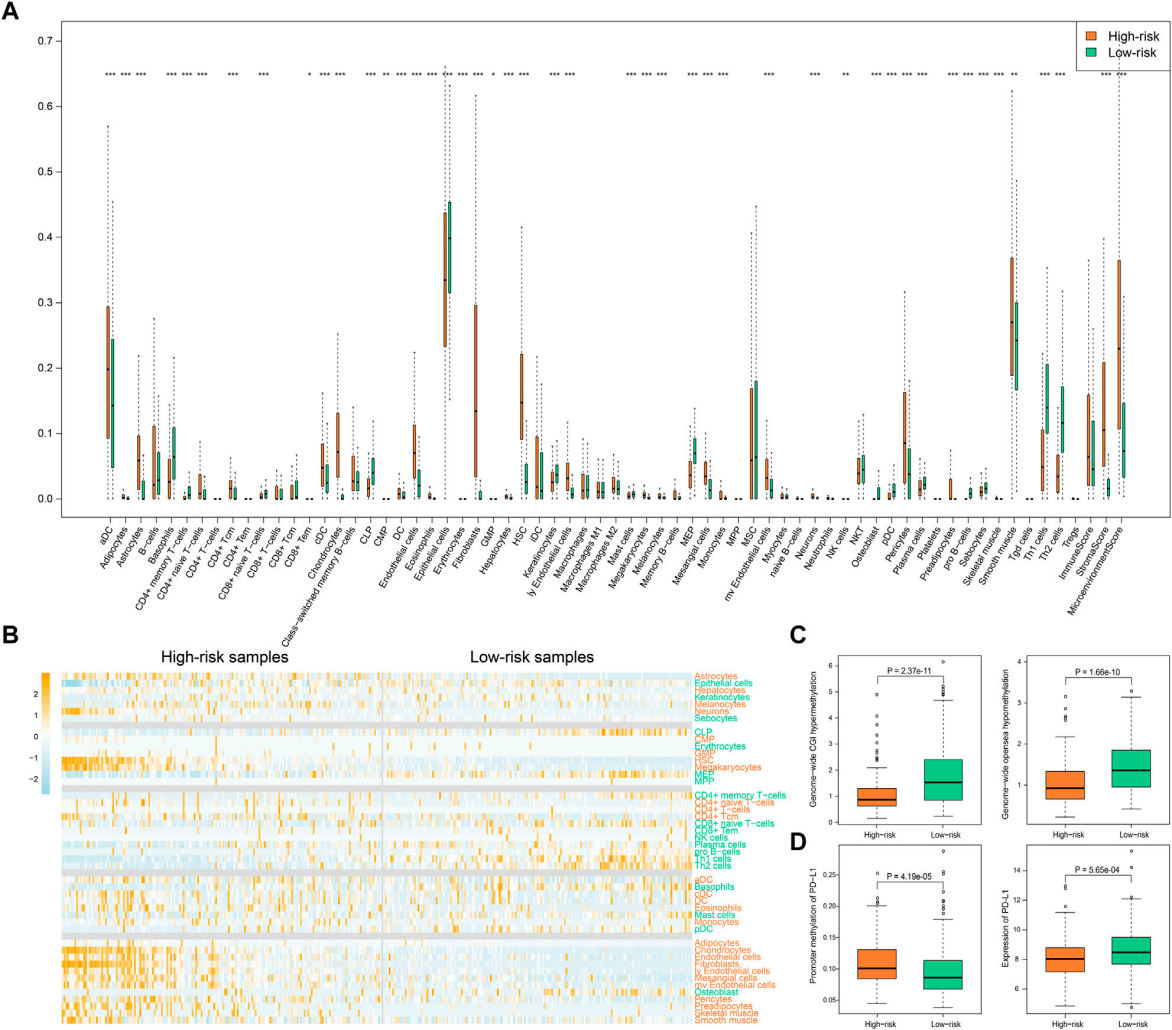
Immune-related score by “xCell”. **(A)** The infiltration of immune and stromal cell types as well as the immune-related scores in high-risk and low-risk samples. *p* values less than 0.05, 0.01, and 0.001 are marked with “*”, “**”, and “***”. **(B)** The heatmap of infiltration degree of those immune and stromal cell types with significantly different infiltration in high- and low-risk samples. Cell types marked with orange color represent higher infiltration in high-risk samples while cell types marked with green color represent higher infiltration in low-risk samples. Samples are sorted by SSA-NHEJ score. **(C)** Genome-wide hypermethylation in CGI regions and hypomethylation in open sea regions. **(D)** Promoter methylation and expression of PD-L1 in high-risk and low-risk samples. *p* values were evaluated by Wilcoxon test.

We further compared the enrichment levels of different types of cells from gene expression data for 64 types of immune and stromal cells in the two types of samples (Figure 7A, Supplementary Table S3). The results showed that the low-risk samples had more infiltrating adaptive immune cells including CD8^+^ TEM cells, CD8^+^ naive T cells, CD4^+^ TCM cells, Th1 cells, and Th2 cells, and the scores of infiltration immune cells were also higher, which might be a key factor for favorable prognosis of the low-risk samples. Recent studies have shown that CD8^+^ T cells are regarded as the main driver of anti-tumor immunity (Reiser and Banerjee, 2016; Fang et al., 2020). Additionally, we found infiltration scores of stromal cells, such as endothelial cells, fibroblasts, and pericytes, were relatively high in high-risk samples compared to low-risk samples. Subsequently, on the basis of data for the high-risk and low-risk samples, we generated a heatmap of immune cells with significant differences and performed a differential analysis of gene expression of the immune checkpoint PD-L1 (Figures 7B–D). Using the HR-LR model, the low-risk samples were associated with a favorable prognosis with a median survival time of 2,197 days, while the high-risk samples had a median survival time of 635 days. The low-risk samples were characterized primarily by infiltration of high levels of CD4^+^ memory T cells, CD8^+^ naive T cells, CD8^+^ TEM cells, natural killer cells, plasma cells, pro-B cells, Th1 cells, and Th2 cells. Subjects in the high-risk samples had shorter overall survival times and exhibited significant increases in the infiltration of CD4^+^ naive T cells, CD4^+^ T cells, CD4^+^ TCM cells, megakaryocytes, dendritic cells, and eosinophils. PD-L1 has been found to be an immune checkpoint. A previous study found that global hypomethylation of DNA could contribute to the upregulation of PD-L1 in melanoma cells and had an impact on DNA repair pathways (Emran et al., 2019). We selected CpG islands and open-sea regions to measure methylation levels in each STAD sample by the method described in the Materials and Methods section. The results showed that high-risk samples had lower methylation levels in CpG islands and higher methylation levels in open-sea regions in comparison with low-risk samples (Wilcoxon *p* = 2.37e−11 and 1.66e−10, respectively; Figure 7C). In addition, high-risk samples were characterized by a significantly lower expression level of PD-L1 (Wilcoxon *p* = 5.65e−04; Figure 7D). This might have been caused by the higher methylation level of CpG sites in the promoter region of the gene encoding PD-L1 (Wilcoxon *p* = 4.19e−05; Figure 7D). The relationships between global methylation, promoter methylation, and expression of PD-L1 and DNA repair processes need to be further studied. Moreover, we also calculated the expression of other immune checkpoints. We found that high-risk samples were characterized by a significantly higher expression level of immune checkpoint genes including CD27, CD40, and CD160. A previous study found that tumor samples with high levels of CD8^+^ tumor-infiltrating lymphocytes were associated with good outcomes in bladder cancer and had significantly higher levels of genes encoding immune checkpoints, such as PD-L1 (Vidotto et al., 2019). Several studies have shown that tumors with a higher TMB can produce more neoantigens, which are more easily recognized by T cells and induce greater immune cytotoxic activity (Rooney et al., 2015). In addition, multiple studies have shown that patients with positive expression of PD-L1 in tumors and infiltration of CD8^+^ T cells have longer overall survival times (Wang et al., 2018; Wang et al., 2021), as was observed in our analysis.

### Cancer-Normal Model Was Developed to Predict the State of an Individual

**FIGURE 8:**
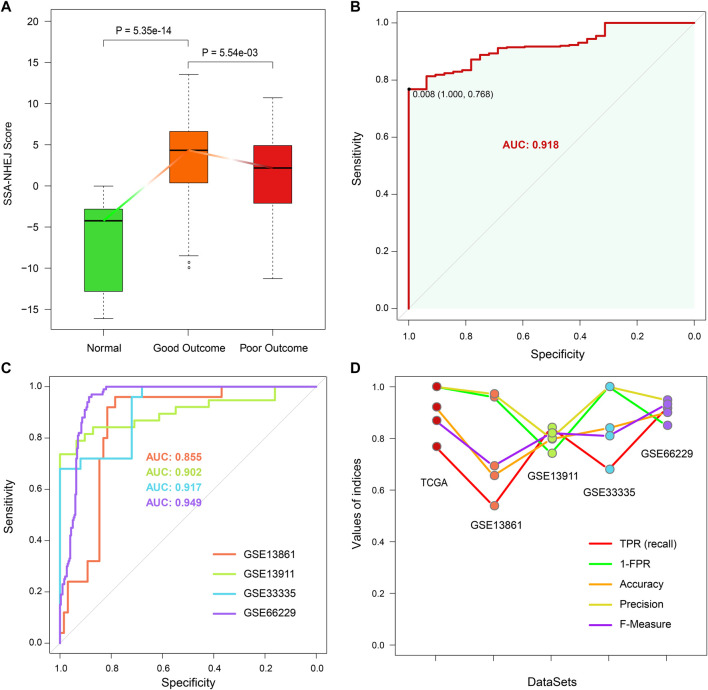
Constructing Cancer-Normal model and predicting the status of samples. **(A)** SSA-NHEJ score of a normal, good outcome, and poor outcome samples. *p* values were evaluated by Wilcoxon test. **(B)** ROC curve of predicting TCGA STAD cancer and normal samples. 0.008 is selected to be the cutoff to predict a sample as normal or cancer status. **(C)** ROC curves of predicting sample status in four independent GEO cohorts. AUC values are listed. **(D)** Five measures of predictive effect for TCGA and four GEO cohorts are listed, including true positive rate, 1—false-positive rate, accuracy, precision, and F-measure.

As described in the first Results subsection, we found that SSA and NHEJ have low activity in normal cells because less replication occurs in normal cells in comparison with cancer cells. As the SSA-NHEJ score is related to the activity of the SSA and NHEJ repair mechanisms, we inferred that normal samples should have lower SSA-NHEJ scores in comparison with cancer samples. This hypothesis was validated, as shown in Figure 8A. Normal samples had the lowest SSA-NHEJ scores, while cancer samples associated with good outcomes had the highest SSA-NHEJ scores (Figure 8A; all *p* < 0.05). From the receiver operating characteristic plot, we selected 0.008 as the cut-off value for distinguishing cancer samples from normal samples, as 0.008 corresponded to high sensitivity and specificity (Figure 8B; AUC = 0.918). The forecasting performance of the Cancer–Normal model was validated in four other independent datasets obtained from the GEO database (Figure 8C; AUC = 0.855, 0.902, 0.917, and 0.949 for the GSE13861, GSE139911, GSE33335, and GSE66229 datasets, respectively). Five measures were utilized to evaluate the performance of the Cancer–Normal model, namely, the true positive rate (or sensitivity), 1—the false positive rate (or specificity), accuracy, precision, and the F-measure. All these measures showed that the Cancer–Normal model gives good predictions of the status of individuals (Figure 8D).

### Evaluation of the HR-LR and Cancer-Normal Models

**FIGURE 9:**
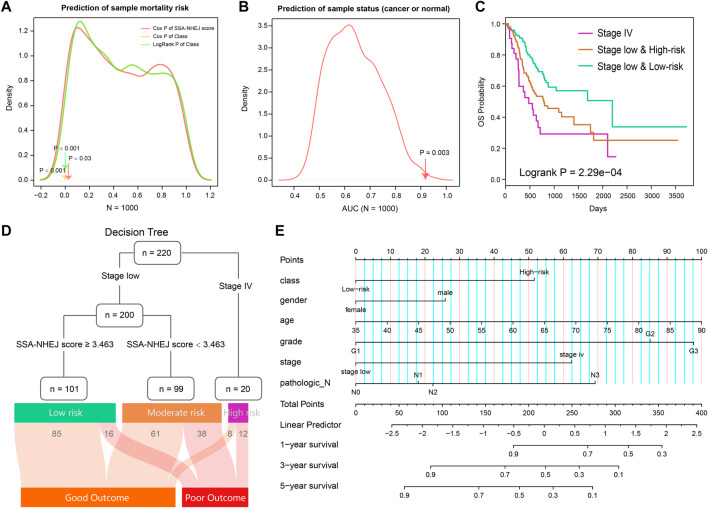
Evaluation of two models and combing clinical features to predict risk assessment for individuals. **(A)** The prognosis of 1000 random HR-LR models based on the expression of random gene sets. **(B)** AUC values of 1000 random Cancer-Normal models based on the expression of random gene sets. **(C)** Survival plot of TCGA STAD patients classified by stage and SSA-NHEJ score. **(D)** Decision tree to predict the patient's clinical outcome. The result of samples in the TCGA cohort is listed at to bottom. **(E)** A nomogram plot is constructed to quantify risk assessment for an individual patient.

The prognostic effect of the HR-LR model and the predictive accuracy of the Cancer–Normal model with regard to cancer or normal health status were validated in the TCGA cohort and various GEO cohorts. We then evaluated these two models by comparing their predictive accuracy with that of random models. By randomly selecting 76 genes from the expression profiles, computing random scores, and selecting a cut-off as in the case of the real models, we constructed a random HR-LR model and Cancer–Normal model. By repeating this process 1000 times, we obtained 1000 random HR-LR models and Cancer–Normal models. We compared the prognostic effects of the random HR-LR models with that of the real HR-LR model. The results showed that the SSA-NHEJ score and the derived class (high-risk vs. low-risk class) were significantly more accurate prognostic factors (Figure 9A; *p* = 0.03, *p* < 0.001, and *p* < 0.001 for univariate Cox *p* of SSA-NHEJ score, univariate Cox *p* of class and log-rank *p* of class, respectively). On the other hand, we compared the AUC values of the 1000 random Cancer–Normal models with that of the real Cancer–Normal model. The results showed that the SSA-NHEJ-related Cancer–Normal model had a significantly higher AUC value (Figure 9B; *p* = 0.003). Together, these results validated the effectiveness of our HR-LR model and Cancer–Normal model and confirmed that their predictive accuracy was not randomly achieved.

From previous results, we found that the patient disease stage also had a significant prognostic value as well as the SSA-NHEJ score (Figure 3A). We, therefore, combined the HR-LR model and patient disease stage and obtained three classes of samples with more significantly different clinical outcomes (Figure 9C; log-rank *p* = 2.29e−04). On the basis of this result, we constructed a decision tree to help predict the clinical outcome for an individual (Figure 9D). In addition, a nomogram was constructed to quantify the survival probability for individual GC patients (Figure 9E). The SSA-NHEJ-derived class (high-risk or low-risk) and several clinicopathological features were included in the nomogram, such as age, gender, disease stage, and disease grade. The C-index reached 0.702 after 1000 bootstrap iterations in the TCGA cohort (0.734 and 0.728 for the training and test sets, respectively). The calibration curve also indicated good agreement between the estimates and observations, which suggested that our nomogram had a high level of accuracy (Supplementary Figure S2). The decision tree and nomogram could contribute to the prognosis in the case of an individual.

## Discussion

DNA repair is a vital biological process in normal physiological conditions and includes various types of repair approaches, such as base excision repair, mismatch repair, and DSB repair. Some repair methods could reduce the number of mutations in individuals, while other repair approaches such as SSA and NHEJ might result in fairly large errors and lead to the accumulation of many somatic mutations (Negrini et al., 2010; Lu et al., 2014). In this study, we extracted GO processes and pathways related to DNA repair from the MSigDB. By combining clinical data from patients in the TCGA STAD cohort, we found that clinical outcomes in GC patients were significantly associated with DSB repair and were especially strongly correlated with the SSA and NHEJ approaches. By the Pearson correlation test, we selected 76 genes with significant correlations with the ssGSEA scores of SSA and NHEJ. These 76 genes comprised 37 positively correlated genes and 39 negatively correlated genes, which were further subjected to the *t*-test to obtain the value of the *t*-statistic for each sample. We referred to the value of the *t*-statistic as the SSA-NHEJ score. Follow-up analyses showed that the SSA-NHEJ score was a valuable prognostic factor for overall survival and recurrence in GC patients. In addition, the SSA-NHEJ score could also predict whether an individual had GC.

Numerous studies have demonstrated that the SSA and NHEJ approach in DSB repair would lead to the accumulation of somatic mutations in comparison with homologous recombination repair (Turner et al., 2004; Yang et al., 2011). In our study, we further confirmed this conclusion. Samples with higher SSA and NHEJ activities (or ssGSEA scores) were found to have higher TMBs. On the other hand, we found that SSA and NHEJ activities were significantly higher in cancer samples with good outcomes in comparison with normal samples, but the increase was significantly less in cancer samples with poor outcomes. On the basis of this phenomenon, we suggest that in normal physiological conditions biological activities such as DNA replication are maintained within normal limits, and hence the SSA and NHEJ approaches are not much needed. However, in cancer cells, DNA replication and other activities increase rapidly, which leads to increases in the activities of the SSA and NHEJ processes. On the other hand, the higher activities of SSA and NHEJ and the higher TMB would lead to the apoptosis of cancer cells (Lu et al., 2014; Hu et al., 2019) or the production of new antigens and finally result in good outcomes in patients (Klempner et al., 2020). In contrast, in other GC patients lower activities of SSA and NHEJ finally, lead to poor outcomes.

There have also been many studies that focused on the relationship between TMB and immune infiltration. Some studies found that a higher TMB is associated with more immune infiltration, while some studies obtained the opposite result. In this study, low-risk samples were found to have a higher TMB but less infiltration of general immune cells. However, significantly higher abundances of CD8^+^ T cells and CD4^+^ memory T cells were observed in low-risk samples. In addition, we found that PD-L1 had higher expression levels in low-risk samples. Similarly, other investigators also found that CD8 positivity is significantly associated with PD-L1 expression (Vidotto et al., 2019). The relationship between the TMB and immune infiltration and their roles in GC needs to be further investigated.

The HR-LR model was constructed on the basis of the training set of sequencing data from the Illumina platform. The predictive accuracy of the model was validated in the test set, which also comprised sequencing data. Furthermore, the prognostic value of the HR-LR model was validated in several GEO datasets, which contained microarray data from different platforms. Similarly, the Cancer–Normal model was also constructed on the basis of Illumina sequencing data and validated in microarray data from different platforms. In addition, we validated the accuracy of the two models by comparing them with random models. These results proved that our HR-LR model and Cancer–Normal model had stable accuracy and could be used universally on different platforms. Combining the models with clinical features will contribute to the prognosis in GC patients.

In summary, we found that SSA and NHEJ are vital prognostic factors in GC, proposed two models to help predict clinical outcomes in GC patients, and investigated the relationships among the SSA and NHEJ approaches, the TMB and immune infiltration, and their roles in GC. The present study aims to provide an improved understanding of the complexity of DNA repair, the TMB, and immune infiltration in GC and to contribute to the development of clinical diagnosis and treatment.

## Conclusion

In summary, we found that SSA and NHEJ were the most prognostically effective DNA repair processes in GC patients. On the basis of the activities of these two approaches and expression profiles, in this study, we proposed two models to help predict clinical outcomes in GC patients and investigated the relationships among the SSA and NHEJ approaches, the TMB and immune infiltration, and their roles in GC. Moreover, we estimated methylation levels in each STAD sample. The present study aims to provide novel insights for the understanding of the complexity of DNA repair, the TMB, and immune infiltration in GC and for further investigation of their diagnostic value.

## Data Availability

The datasets presented in this study can be found in online repositories. The names of the repository/repositories and accession number(s) can be found in the article/Supplementary Material.
