# Supplementary figures and images for: Analysis of DNA Repair-Related Prognostic Function and Mechanism in Gastric Cancer
Source: Front Cell Dev Biol. 2022 May 17;10:897096. doi: 10.3389/fcell.2022.897096 (PMC9152153; doi:10.3389/fcell.2022.897096)

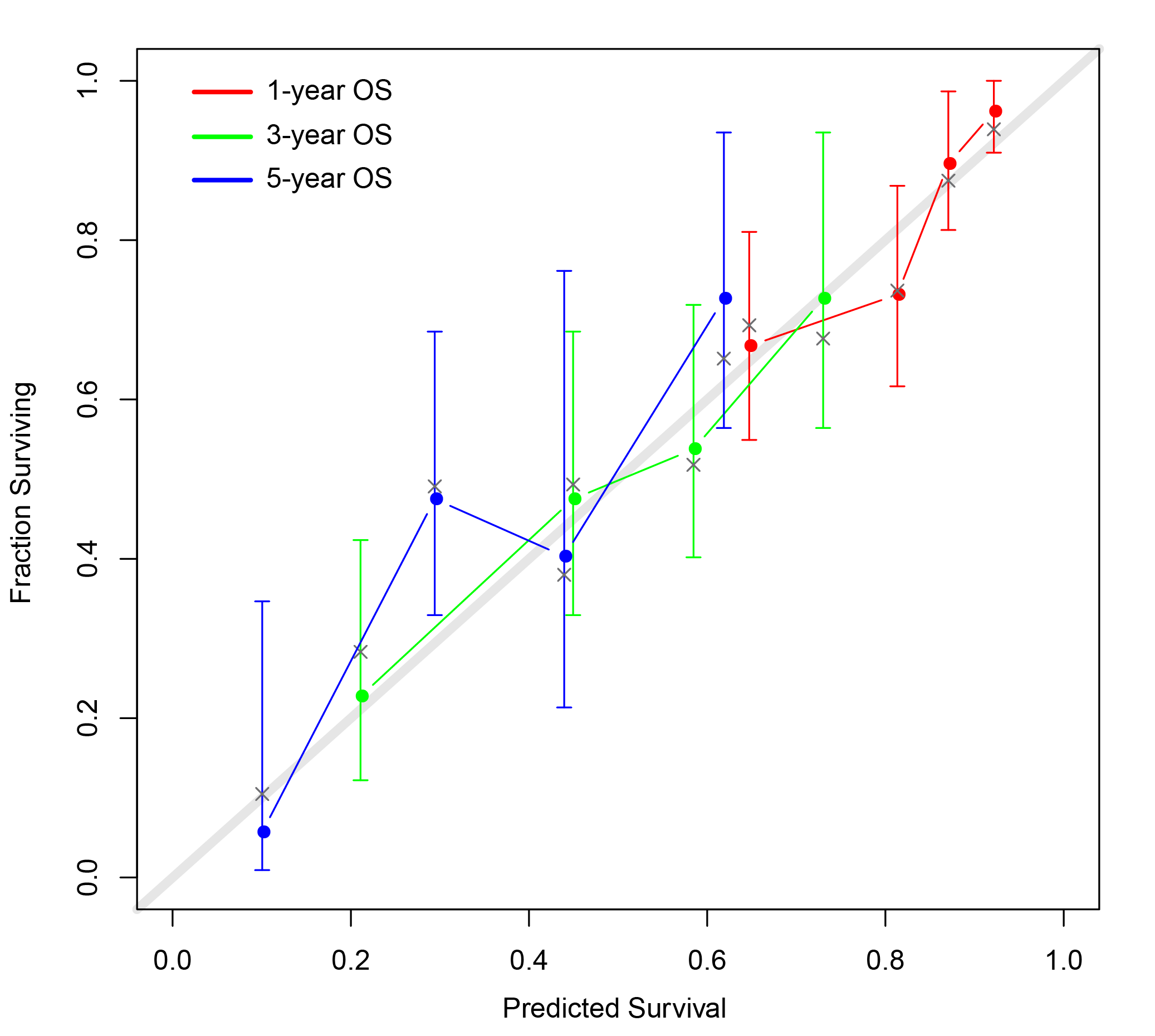

Supplement: Supplementary file 3 [file Image2.TIF]

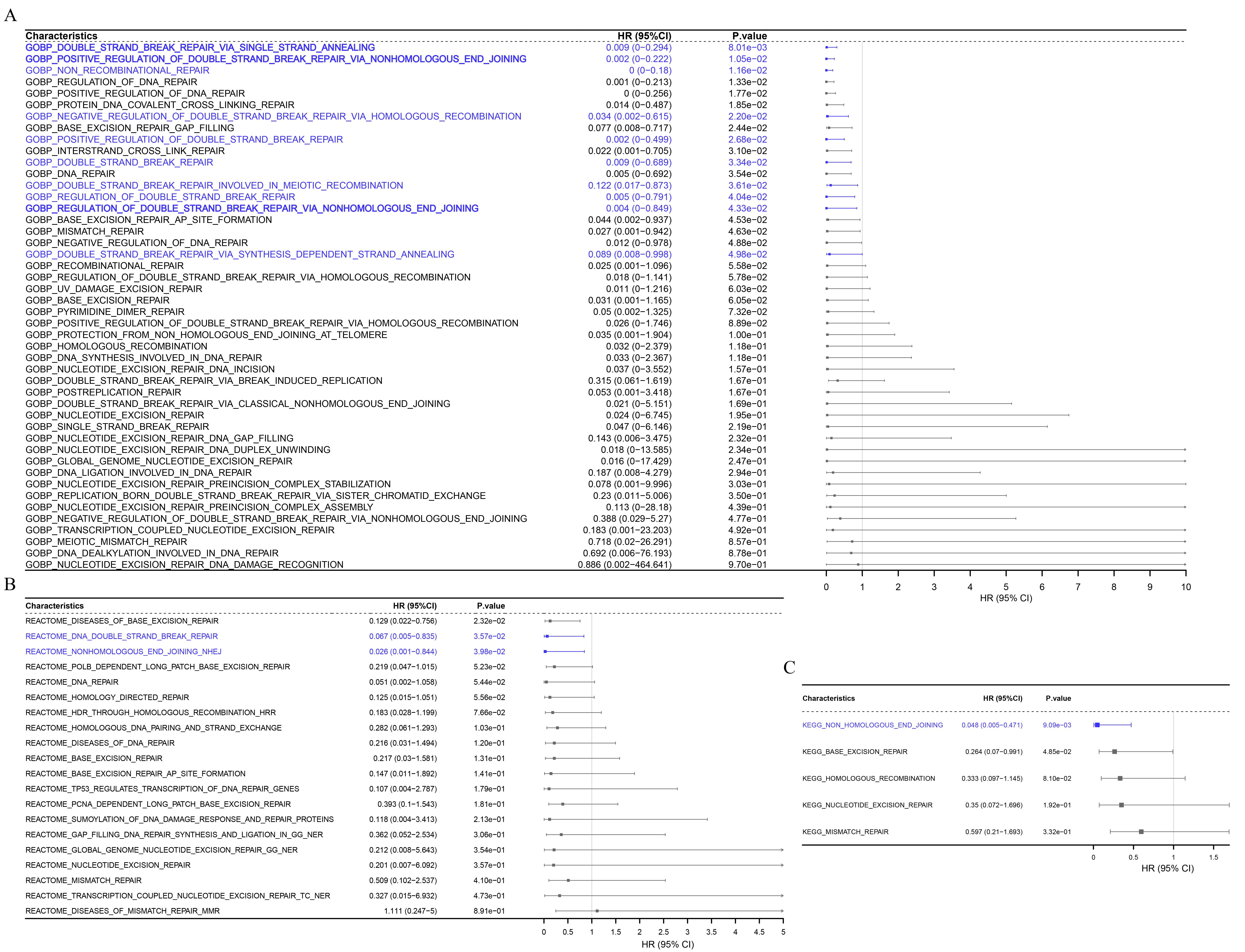

Supplement: Supplementary file 4 [file Image1.TIF]
